# The Use of Non-Conventional Yeast in Sake Production

**DOI:** 10.3390/molecules30183786

**Published:** 2025-09-18

**Authors:** Agnieszka Wilkowska, Zuzanna Dzwonnik

**Affiliations:** Institute of Fermentation Technology and Microbiology, Faculty of Biotechnology and Food Science, Łódź University of Technology, Wólczańska 171/173, 90-924 Łódź, Poland

**Keywords:** non-conventional yeast, sake aroma, sake quality, ethyl carbamate precursors

## Abstract

In response to the growing interest in less conventional alcoholic beverages, this study aimed to identify novel yeast strains suitable for sake production, with a focus on their potential application in bioflavouring. Commercially available strains of bottom-fermenting brewing yeasts (*Saccharomyces pastorianus*), a cryotolerant wine yeast (*Saccharomyces bayanus*), and a wild wine yeast (*Torulaspora delbrueckii*) were evaluated. The quality characteristics of sake obtained using non-conventional yeasts were compared with sake produced using *Saccharomyces cerevisiae* K7, one of the most commonly used strains in sake brewing. Sake made with non-conventional yeasts exhibited differences in fermentation kinetics, chemical composition, and sensory properties. Wine yeasts produced sake with the most favorable ester profile, markedly distinct from those obtained with other yeast strains used in the study. Compared to the conventional strain, the concentrations of the key contributors to the fruity/floral aroma, namely 3-methylbutyl acetate and ethyl hexanoate, in sake produced with *S. bayanus* were higher by 249.5% and 199.3%, respectively. The wine yeast *S. bayanus* may be considered the most promising strain for sake production due to its ability to generate elevated levels of volatile aroma compounds associated with Ginjo-ka characteristics, as well as its effectiveness in supporting a consistent and efficient alcoholic fermentation process.

## 1. Introduction

Sake is a traditional Japanese alcoholic beverage made from steamed rice, water, yeast, and koji. Its production involves a unique process in which saccharification and fermentation occur simultaneously. Unlike beer brewing, where fermentation takes place after mashing, sake brewing employs a parallel double fermentation. In this process, starch from rice is hydrolyzed by enzymes produced by the mold *Aspergillus oryzae* present in koji, while the resulting sugars are fermented by sake yeast.

Rice serves as the primary raw material in sake production. The rice polishing ratio and the addition of alcohol are the main criteria used to classify different types of sake. Unlike grapes, whose varietal aromas strongly shape the flavor of wine, rice has a low aromatic potential and therefore exerts only a limited influence on the aroma of sake. By definition, sake cannot contain any added ingredients intended to enhance aroma or flavor, which highlights the crucial role of microorganisms in shaping its sensory characteristics. Among these, yeast is considered the most important factor influencing the aromatic profile of sake [1,2,3,4,5,6].

Yeasts of the species *Saccharomyces cerevisiae* are among the most important industrial microorganisms used for ethanol fermentation. Similarly to wine, beer, and spirit production, the yeasts used in sake brewing are also classified as *S. cerevisiae*. However, these microorganisms exhibit several distinct characteristics that make them particularly suitable for sake fermentation. These include adaptation to lower fermentation temperatures, high ethanol tolerance, low foam formation, reduced urea and pyruvate production, and high ester productivity (e.g., isoamyl acetate and ethyl caproate), among others [7,8].

In recent decades, the use of *S. cerevisiae* Kyokai no. 7 (K7) group strains, represented by K6, K7, K9, K10, and their non-foaming derivatives, has become a common practice in sake production. The K7 strain is characterized by high ethyl caproate production, strong fermentation performance, and excellent brewing quality at low temperatures (10–12 °C). Strains K6 and K9 additionally produce elevated levels of isoamyl acetate, contributing to the fruity and refreshing aroma of sake. The K10 strain is known for yielding sake with lower acidity. Strains K-601, K-701, and K-901 are classified as non-foaming yeasts. These strains are officially provided by the Brewing Society of Japan for use in sake production, owing to their unique properties and proven compatibility with the brewing process [6]. Only small amounts of sake are brewed using unique house sake yeast typical for the brewery. Although this approach positively influenced the overall quality of sake produced on an industrial scale, the diversity of its sensory properties declined. Therefore, a trend in brewing sake with yeast other than K7 group strains emerged [9].

During alcoholic fermentation, yeasts are responsible not only for ethanol production but also for the synthesis of numerous flavour-active compounds that contribute to the characteristic aroma and taste of fermented beverages. Sake yeasts produce a variety of metabolites, including volatile compounds (e.g., esters, higher alcohols, carbonyl compounds) and non-volatile compounds (e.g., glycerol, organic acids), which are key contributors to the flavor and taste of sake. Thus, employing yeast strains that differ metabolically from the conventional K7 strain can change the composition of flavor-active compounds and increase the diversity of sake styles and products [10,11].

To obtain sake yeast strains with distinct metabolic capabilities, selective breeding has proven to be an effective strategy. Several approaches have been applied, including mutagenesis, cell fusion, and crossbreeding [12]. Within the sake brewing industry, particular emphasis has been placed on developing yeast mutants that: (1) produce elevated levels of desirable flavor compounds (e.g., isoamyl acetate, ethyl caproate, β-phenethyl alcohol) while reducing the formation of off-flavors such as dimethyl trisulfide [13,14,15,16,17]; (2) do not form foam [18,19]; (3) do not generate urea, a precursor of the carcinogenic compound ethyl carbamate [20]; (4) exhibit high ethanol tolerance [21]; (5) accumulate elevated concentrations of malate [22,23,24]; (6) overproduce tyrosol [25,26]; or (7) enhance peptide production [27]. The publication of genome sequences for sake yeasts has facilitated the identification of quantitative trait loci (QTLs) associated with key brewing characteristics, enabling the application of DNA marker–assisted selection in mated strains [8,28,29]. In parallel, recent advances in genetic engineering have been utilized to improve sake yeast strains. For example, Chadani et al. [30] employed a three-step serial breeding approach combined with an optimized CRISPR–Cas9 genome-editing system to introduce eight targeted mutations into a standard sake yeast strain. The resulting variant lacked foam formation, produced sake free of carcinogenic compounds and unpleasant sulfurous notes, and generated a characteristic sweet ginjo aroma. Furthermore, sake yeasts that undergo genetic modification followed by the removal of extraneous DNA sequences are classified as self-cloning yeasts. Importantly, under Japanese government guidelines for GMO foods, such strains are not considered genetically modified organisms [8,31].

Beyond laboratory-bred strains, a number of useful natural sake yeast isolates have also been obtained from brewing facilities across different regions of Japan [9,32]. On the other hand, certain non-conventional yeast species have recently been proposed as starter cultures in brewing and winemaking to create unique and high-quality products. These include genera such as *Pichia*, *Saccharomycodes*, *Zygosaccharomyces*, *Scheffersomyces*, *Hanseniaspora*, *Torulaspora*, and *Lachancea*. Selected strains of these non-conventional yeasts can positively influence beverage quality by enhancing aroma complexity, increasing flavor diversity, and contributing to the production of other metabolites that affect the character and style of the final product, such as ethanol content, acidity, glycerol, mannoproteins, and color stability [33,34,35].

Bioflavouring represents a natural strategy for enhancing the aroma profile of alcoholic beverages through the use of non-conventional yeast strains during fermentation. Due to high need for various types of yeast strains in the sake industrial sector, this study aimed to evaluate non-conventional yeast strains for sake production during the simultaneous saccharification and fermentation process (SSF). Commercially available strains of bottom-fermenting brewing yeast (*Saccharomyces pastorianus*), cryophilic wine yeast (*Saccharomyces bayanus*), and wild wine yeast (*Torulospora delbrueckii*) were evaluated. The quality characteristics of sake obtained using non-conventional yeast were evaluated by comparison with wine produced using one of the most popular yeast strains for sake production *Saccharomyces cerevisiae* K7. A comprehensive analysis of the obtained sake was conducted, covering the dynamics of fermentation process, basic chemical composition, ethyl carbamate and its precursors, profile of volatile by-products of alcoholic fermentation, antioxidant activity, color, and sensory characteristic.

## 2. Results and Discussion

### 2.1. Alcoholic Fermentation Kinetics and Basic Chemical Composition of Sake

**Figure 1:**
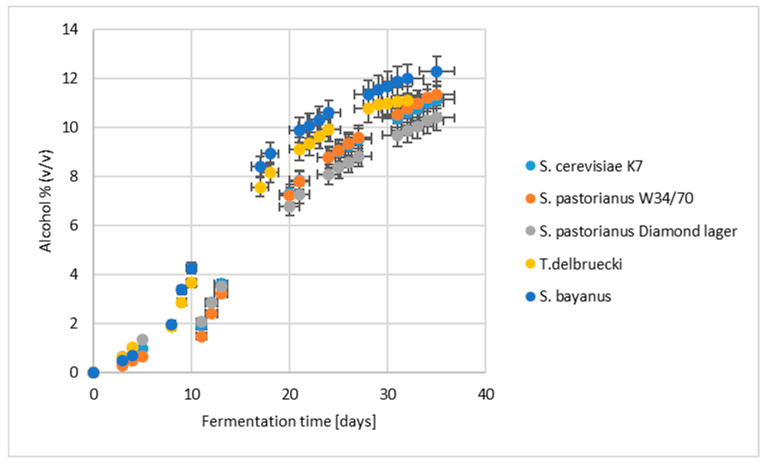
The kinetics of alcoholic fermentation of sake inoculated with different yeast species.

Both conventional and non-conventional yeast strains used for moromi inoculation exhibited appropriate alcoholic fermentation dynamics, reaching ethanol levels required for sake production (Figure 1). The fermentation profile of the conventional *S. cerevisiae* K7 strain closely resembled that of the brewing yeast *S. pastorianus* W34/70 throughout the process. In contrast, fermentation with *S. pastorianus* Diamond Lager showed slower kinetics, with a delay of approximately five days. Such delays are relevant at the industrial scale, as they may prolong production time and increase resource consumption. Conversely, the cryotolerant wine yeast *S. bayanus* demonstrated the highest fermentation efficiency, achieving elevated ethanol concentrations within the shortest time frame.

Sake production in Japan is regulated by the National Tax Agency. Under Japanese liquor tax regulations, sake is required to contain less than 22% alcohol by volume; however, the typical content on the label is around 15% (*v*/*v*). Our findings show that all products obtained in this study meet the required criterion and can be classified as low-alcohol sake. The level of produced alcohol ranged from 10.7 to 12.3% (*v*/*v*) for *S. pastorianus* Lager and *S. bayanus*, respectively (Table 1). Variability in ethanol yield among yeast strains has been attributed to differences in sugar uptake and metabolism [36,37], nitrogen assimilation [38,39], ethanol tolerance [40,41], redox balance through by-product formation such as glycerol [42,43], and genetic regulation of alcohol dehydrogenase expression [44].

The average content of total acidity in sake ranges between 1.1 and 2.4 g/L. However, recent research conducted by Nakatani et al. [45] on the acidifying potential of *L. thermotolerans* strains in sake brewing, reported much higher concentration—3500 ppm of lactic acid and an ethanol content of approximately 11–12% (*v*/*v*), recognizing high suitability of this strain for sake production. The level of total acidity in sake analysed in our study varied notably depending on the yeast strain used for fermentation (Table 1). *T. delbrueckii* fermentation led to significantly higher values (2.42 g/L) compared to all other strains, indicating more intense acid production. Similarly, volatile acidity was the greatest in sake fermented with *T. delbrueckii* (0.54 g/L) and *S. bayanus* (0.50 g/L), which could influence the aromatic and flavor profile of the final product. In contrast, the lowest volatile acidity was observed in *S. pastorianus* W34/70 (0.33 g/L), potentially contributing to a cleaner sensory profile. Volatile acidity in sake primarily consists of acetic acid and, to a lesser extent, other short-chain fatty acids. It serves as an important quality indicator, as elevated levels (approximately above 1 g/L) may signify microbial spoilage or deviations in the fermentation process.

Regarding amino acidity (Table 1), the highest values was recorded for *S. cerevisiae* K7 (2.55 mL), *S. pastorianus* W34/70 (2.45 mL) and *T. delbrueckii* (2.20 mL) suggesting enhanced proteolytic activity or amino acid release, which may contribute positively to umami and complexity in sake flavor. The lowest amino acidity was observed in both *S. pastorianus* Diamond Lager and *S. bayanus*, indicating reduced nitrogenous compound formation. An increase in amino acidity generally enhances the taste of sake, imparting richness, body, and savory depth. However, excessively high levels may result in a heavy or flat flavor, whereas insufficient amino acidity can lead to a lack of complexity. The perception of umami is driven primarily by free glutamate and, to a lesser extent, by free aspartate [46]. Schmidt et al. [47] reported that not only sake, but also certain beers, wines, and champagnes that undergo extended yeast contact, contain notable concentrations of free glutamate capable of eliciting umami taste. Peptides and free amino acids (FAAs), which contribute to flavor development, may originate either from the rice substrate or from microbial autolysis, depending on the brewing process. Premium sake types such as Ginjo and Daiginjo, brewed from highly polished rice, typically exhibit lower amino acidity, resulting in a lighter sensory profile. Nevertheless, amino acid levels in sake are influenced not only by the polishing ratio, but also by the yeast strain and the activity of specific bacteria, particularly lactic acid bacteria [48]. Beyond their role in umami perception, amino acids also modulate the balance between sweetness and bitterness. Iwano et al. [49] identified alanine as a contributor to sweetness, whereas arginine was associated with bitterness. Amino acidity further affects the aging potential of sake through participation in the Maillard reaction, a complex chemical process in which nucleophilic groups from amino acids, peptides, and proteins react with carbonyl groups from reducing sugars. This reaction generates a wide range of volatile compounds and brown pigments known as melanoidins [50]. As a result, sake with higher amino acidity tends to undergo more pronounced maturation, developing complex flavor attributes such as nuttiness and caramel through Maillard-derived pathways [51,52].

Although no external sulfites are added to some wines, they may still contain measurable amounts of sulfites produced endogenously by fermenting yeasts as a natural by-product of the fermentation process. In yeast metabolism, sulfur dioxide (SO_2_) acts as an intermediate in the biosynthesis of sulfur-containing amino acids such as methionine and cysteine. It is generated through the sulfate reduction pathway, which is activated when there is a cellular demand for these amino acids, compounds often limited in fermenting wort. Under conditions that lead to significant intracellular accumulation of sulfite ions (SO_3_^2−^), yeast cells activate protective mechanisms involving active transport systems that export excess sulfite into the extracellular environment. This process results in an increased concentration of sulfur dioxide in the fermentation medium, such as wine and beer [53,54,55]. Additionally, sulfites act as precursors to hydrogen sulfide, an undesirable by-product due to its detrimental effect on wine aroma. Consequently, elevated concentrations of SO_2_ in wine are regarded as unfavorable. Ogata et al. [56] identified a relationship between nitrogen deficiency and increased hydrogen sulfide (H_2_S) production by lager yeast, which may also indirectly contribute to elevated sulfur dioxide levels. The endogenous production of sulfur dioxide during alcoholic fermentation is governed by multifactorial interactions involving genetic determinants, metabolic pathways, and physicochemical conditions, with key contributions from the yeast strain employed and the chemical composition of the must.

In this study, several yeast strains commonly used in winemaking and brewing were employed as non-conventional microorganisms for sake production. Although these strains were selected by the manufacturers based on fermentation-related properties, including low SO_2_ production, the composition of the must, specifically using rice wort instead of wine must or brewing wort, could significantly influence SO_2_ levels. However, all yeast strains produced low concentrations of free (2.30–2.83 mg/L) and total SO_2_ (13.49–14.07 mg/L) (Table 1), demonstrating their suitability for use in the sake production process with respect to SO_2_ levels. According to Zhang et al. [57], sulfur dioxide levels in lager beer under different wort oxygenation conditions ranged between 14 and 18 mg/L. In turn, wine yeasts are capable of producing anywhere from a few milligrams per liter of sulfites to over 90 mg/L [58,59]. Considering that a small proportion of the population is sensitive to sulphites (manifesting symptoms such as dermatological reactions, respiratory distress, gastrointestinal disturbances, and cardiovascular effects), regulations require disclosure of sulphur dioxide content on the label of alcoholic beverages. This applies when the total concentration of sulphur dioxide equals or exceeds 10 mg/L [60,61].

There have been numerous reports on the antioxidant capacity and associated antioxidant components of sake [62,63,64]. According to the literature, the primary contributors to the antioxidant capacity of sake include phenolic compounds (such as tannic acid, ferulic acid, and p-coumaric acid), tyrosol, peptides, amino acids, and melanoidins. This study also examined the influence of the yeast strains used on the biological activity of the final sake product. The antioxidant activity against the ABTS radical ranged from 0.23 to 0.32 mM/L (Table 1). The results indicate that the type of yeast employed in the sake production process did not significantly affect its radical scavenging activity.

### 2.2. Ethyl Carbamate and Its Precursors

The World Health Organization (WHO) International Agency for Research on Cancer working group classified ethyl carbamate (EC) as a group 2A carcinogen, i.e., probably carcinogenic to humans [65,66]. It is important to note that the presence of ethyl carbamate in fermented foods is of considerable concern. Consequently, the wine industry has shown strong interest in developing effective strategies to reduce EC levels in final products.

Ethyl carbamate is primarily formed through the reaction of ethanol with urea or other carbamoyl-containing compounds, with urea being the main precursor in fermented products. In contrast, elevated EC levels in stone fruit spirits are mainly attributed to cyanate [67,68]. In alcoholic beverages, the main pathway for EC formation involves the yeast-mediated conversion of arginine to urea during early fermentation. Bacterial conversion of arginine to citrulline may also contribute to ethyl carbamate formation through reaction with ethanol. Although less common than the urea pathway, ethyl carbamate formation via citrulline has been observed in red and white table wines, yellow rice wine, and fermented soybean products [69,70].

Sake samples obtained in the course of this study were subjected to analysis for ethyl carbamate precursors, including arginine and urea. The conducted analyses did not confirm the presence of L-arginine in any of the rice wine samples examined, including both conventional and non-conventional varieties. Arginine is classified as a Group A amino acid, which is preferentially consumed by yeast during the early stages of fermentation, thereby explaining its absence in the final alcoholic products [71].

It has been reported that up to 90% of ethyl carbamate in Chinese yellow rice wine originates from the reaction of urea with ethanol [72]. Yeast strains differ in their urea production capacity, the proportion of urea excreted into the medium, and the extent to which it is reabsorbed and utilized. In our study, various *Saccharomyces* and non-*Saccharomyces* yeast strains exhibited differing capacities for nitrogen compound metabolism, which directly influenced the residual urea concentrations in sake after fermentation. The bottom-fermenting brewing yeast *S. pastorianus* and the cryophilic wine yeast *S. bayanus* were generally associated with approximately 36% lower urea production compared to the conventional sake yeast strain. K7, *T. delbrueckii*, and *S. cerevisiae* Lager strains showed no statistically significant differences in urea concentrations (Table 2). In the context of using non-*Saccharomyces* yeasts, Wu et al. [73] demonstrated that *Wickerhamomyces anomalus* contributes more significantly to urea formation in a model system simulating Chinese liquor fermentation, producing considerably higher urea levels (910.0 μg L^−1^) compared to *S. cerevisiae* (300.1 μg L^−1^). In contrast, Benito et al. [74] utilized *S. pombe* instead of *S. cerevisiae* for red wine fermentation, achieving a final urea concentration of less than 0.5 mg/L.

The urea concentration found in sake (4.23–6.62 mg/L) aligns with the range observed in wines by Larcher et al. [75], who reported values between 0.12 and 33.6 mg/L (median 0.33 mg/L) per different wine yeast strain. At these urea concentrations, ethyl carbamate (EC) levels ranged from below the detection limit to 13.5 μg/L before storage and up to 14.3 μg/L after 150 days at <12 °C. In our study, ethyl carbamate (EC) was not detected in any of sake samples, which was analysed immediately after the completion of the fermentation process. However, EC formation is influenced not only by the concentration of its precursors, but also increases during storage and is closely associated with external storage-related factors, including duration, temperature, and exposure to UV light [76,77,78].

Risk assessments have prompted various governmental bodies to set permissible limits for ethyl carbamate in alcoholic beverages. EC limit is established under Korean and Japanese food safety regulations, which classify sake and yellow rice wines as fortified wines. For such beverages, an ethyl carbamate concentration of up to 100 µg/L is considered acceptable. The legal basis for this threshold in Japan includes the Food Sanitation Act [79], as well as relevant provisions under the Japanese Agricultural Standards [80] and regulatory updates issued by the National Tax Agency (NTA) [81] concerning the production and labeling of sake as a fortified alcoholic beverage. Also, Canada specifically limits EC concentrations in sake at 200 μg/L [82].

The analytical method applied in our study provided a limit of detection (LOD) of 4.89 µg/L and a limit of quantification (LOQ) of 14.82 µg/L. Given that our LOD and LOQ are significantly lower than these regulatory thresholds, the absence of detectable EC in our samples indicates that the risk of exceeding permissible limits under the tested conditions is minimal.

To minimize ethyl carbamate formation in the final product, some fermentation industries utilize genetically modified or selectively bred yeast strains with reduced capacity for urea production. The use of a commercial genetically modified yeast strain (ECMo01) with improved urea-degrading activity resulted in a reduction in ethyl carbamate concentrations by about 50% in bread and 90% in red wine [83,84]. The use of urease enzymes has been recognized and authorized by the OIV (International Organisation of Vine and Wine) as part of the International Code of Oenological Practices [85].

### 2.3. Aroma of Sake

Yeast are the most important factor determining the aromatic profile of sake. Terms used to describe the aroma of sake, such as fruity, floral, caramel-like, and earthy, reflect the remarkable diversity of its sensory characteristics. Considering the widespread presence of intensely aromatic rice wine on today’s market, it is often mistakenly assumed that it contains added fruits or herbs. However, by definition, sake must not contain any additional ingredients that enhance aroma or flavor, which clearly illustrates the vital role of microorganisms used in its production in shaping its sensory profile.

Yeasts generate a variety of metabolites, including volatile aroma compounds such as esters, higher alcohols, and carbonyl compounds. In this study, the by-products of alcoholic fermentation in sake samples produced with both conventional and non-conventional yeast strains were analysed in terms of their quantitative and qualitative composition.

**Figure 2:**
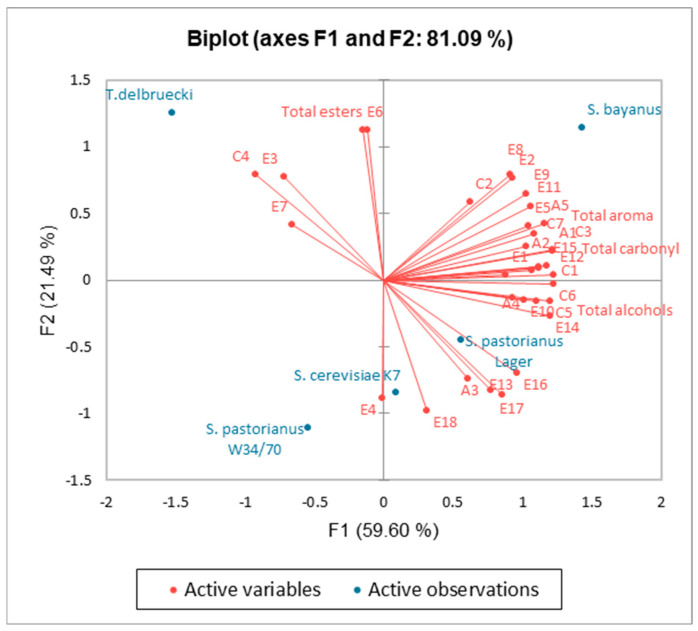
The result of PCA for the composition of volatile fermentation by-products in sake. A1: 1-propanol, A2: Isobutyl alcohol, A3: 3-Methylbutanol, A4: 2-Methylbutanol, A5: 2-Phenylethanol, E1: Methyl acetate, E2: n-propyl acetate, E3: Ethyl isobutyrate, E4: Butyl acetate, E5: Ethyl formate, E6: Ethyl acetate, E7: Ethyl propionate, E8: Isobutyl acetate, E9: Ethyl butyrate, E10: 3-methylbutanol acetate, E11: 2-methylbutanol acetate, E12: ethyl hexanoate, E13: Ethyl heptanoate, E14: Ethyl octanoate, E15: 2-Phenethyl acetate, E16: Ethyl nonanoate, E17: Ethyl decanoate, E18: Ethyl dodecanoate, C1: Acetaldehyde, C2: Acetone, C3: Isobutyraldehyde, C4: 2,3-Butanedione, C5: 3-methylbutanal, C6-2-methylbutanal), C7-Acetal.

A principal component analysis (PCA) was conducted to elucidate similarities and differences in the aroma profiles of standard and non-conventional sake. The analysis encompassed the concentrations of five alcohols, eighteen esters, seven carbonyl compounds, as well as the total concentrations of aroma components, alcohols, esters, and carbonyls (Figure 2). Examination of the explained variance indicated that two principal components accounted for 81.11% of the total variance: the first principal component (F1) explained 59.60%, while the second principal component (F2) accounted for 21.71%. Case classification was performed based on the correlation with each PCA factor.

The PCA results confirmed differences in the aroma profiles among the sake samples investigated. Specifically, four sake samples produced with non-conventional yeasts were distributed across different quadrants of the coordinate system. However, no distinct clustering was observed solely based on yeast type. Notably, sake produced with wine yeast tended to separate along the F2 axis, occupying the first and second quadrants, whereas samples fermented with brewing yeast and sake K7 yeast were primarily located in the third and fourth quadrants. In this context, the attributes positively contributing to wine yeast (*T. delbrueckii* and *S. bayanus*) included total ester content, which was closely associated with ethyl acetate concentration, and additionally ethyl isobutyrate, ethyl propionate, and 2,3-butanedione content. Along PC1, samples were primarily differentiated by their carbonyl compounds, higher alcohols, and total aroma content. Sake produced with Saccharomyces pastorianus Lager yeast exhibited the greatest similarity to conventional sake, as these samples clustered within the same quadrant (IV).

**Figure 3:**
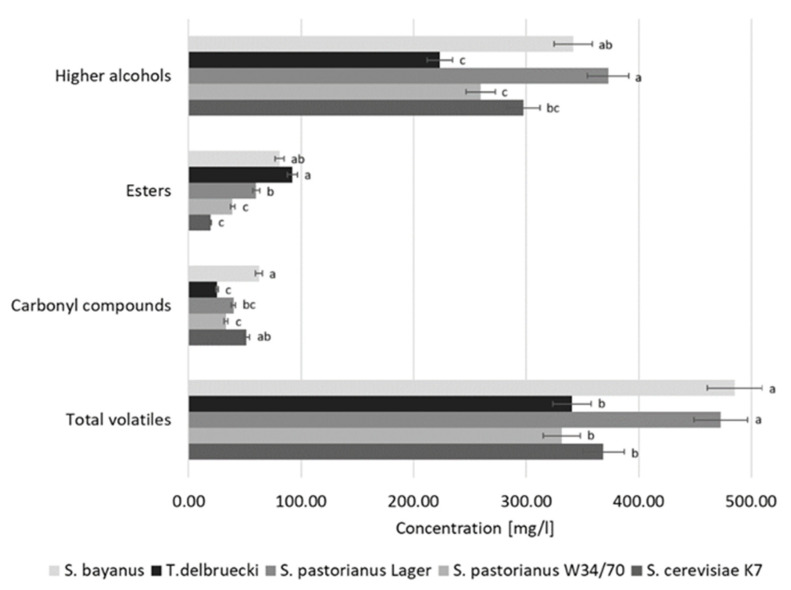
The main groups of volatiles in sake. Data represent means from three replicates of a single experiment. Error bars indicate standard deviations. ANOVA assumptions were verified (Shapiro–Wilk test for normality, *p* > 0.05; Levene’s test for homogeneity of variances, *p* > 0.05). Different letters indicate statistically significant differences between sake variants (ANOVA, *p* < 0.05).

Among sake fermented by different yeast species, the highest concentrations of fermentation by-products were recorded for *S. pastorianus* Lager (472.8 mg/L) and *S. bayanus* (485.3 mg/L), which, compared to the sake based on conventional yeast, constituted 128.5% and 132.7% of the total aromas determined, respectively. There were no significant differences in the total concentration of volatiles between *S. cerevisiae* K7, *S. pastorianus* W34/70, and *T. delbrueckii* (Figure 3).

Compared to ethanol, higher alcohols contain more than two carbon atoms, resulting in increased molecular mass and elevated boiling points. In all types of sake analysed in this study, higher alcohols (fusel alcohols) constituted the largest share of aroma compounds. In sake produced using *S. cerevisiae* K7, fusel alcohols accounted for nearly 81% of the total volatiles, with a concentration of 297.6 mg/L. A similar share of higher alcohols in the aroma of sake was noted for the *S. pastorianus* W34/70 (78%, 259.4 mg/L) and *S. pastorianus* Lager (79%, 372.8 mg/L). In sake samples produced using wine yeast, the percentage of higher alcohols was lower in comparison with conventional sake, amounting to 71% for *S. bayanus* yeast (223.3 mg/L) and 66% for *T. delbrueckii* yeast (341.9 mg/L).

The use of different *Saccharomyces* yeast strains in wine fermentation has been shown to result in significant variations in the levels of higher alcohols present in the final product [86,87]. Among non-*Saccharomyces* yeasts, *T. delbrueckii* is the most extensively studied, commercially available, and widely applied at the industrial scale. However, findings regarding its impact on higher alcohol production remain inconsistent. In sequential fermentations involving three industrial *T. delbrueckii* strains: Zymaflore Alpha (Laffort, Bordeaux, France), BIODIVA (Lallemand, Montreal, QC, Canada), and Viniferm NS-TD (Agrovin Ciudad Real, Spain), only Viniferm NS-TD exhibited the ability to reduce the total concentration of higher alcohols when co-inoculated with *Saccharomyces cerevisiae* [88,89]. In the present study, the total amount of higher alcohols produced by *T. delbrueckii* was approximately 25% lower compared to sake fermented with conventional *S. cerevisiae* K7 sake yeast.

Previous studies have found that higher initial sugar concentrations stimulate yeast to metabolize more alpha-keto acids and enhance amino acid deamidation, which are prerequisites for the production of large amounts of higher alcohols [90]. However, sake is produced through a process of simultaneous saccharification and fermentation, during which the sugar concentration typically does not exceed 240 g/L, which is generally considered a critical threshold for enhanced higher alcohol formation.

Higher alcohols are typically recognized as harsh and pungent; however, at concentrations below 300 mg/L they usually contribute to the desirable complexity of wine and are not regarded as off-flavors [91]. At concentrations above 400 mg/L, the beverage is classified as having poor sensory quality due to its unpleasant and sharp odor. Although sake produced using *S. pastorianus* Lager and *S. bayanus* slightly exceeded the 300 mg/L threshold, these levels did not result in any noticeable sensory defects during the aroma evaluation of those samples.

Esters are considered the most important group of volatile compounds, generally contributing to the fruity and floral aromas in sake. Our results indicated that the wine yeast strains were the most effective ester producers among the tested microorganisms (Figure 3). In comparison with the conventional K7 yeast, the total ester levels in sake fermented with *T. delbrueckii* and *S. bayanus* were approximately 5-fold and 4.3-fold higher, respectively. However, the total ester concentrations detected in all sake samples (ranging from 18.38 to 79.47 mg/L) remain well within the acceptable limits for high-quality beverages, in which total ester concentrations should not exceed 200 mg/L [92]. *T. delbrueckii* contributed positively to the aromatic profile of sake not only by generating increased levels of fruity esters, but also by simultaneously reducing the concentration of higher alcohols. No significant differences in total ester content were observed between sake produced with K7 and *S. pastorianus* W34/70. Nevertheless, the total ester concentration is not the sole determinant of sake aroma. In fact, differences in the qualitative and quantitative composition of individual esters play a key role in defining the unique sensory characteristics of the sake bouquet.

The primary ester found in wine, beer, and sake is ethyl acetate. In the analyzed sake samples, ethyl acetate, present at concentrations ranging from 17.37 to 90.86 mg/L, accounted for a relatively high proportion of total esters (to over 94%)—Table 3. Typical ethyl acetate concentrations in beer range from 8 to 32 mg/L, while in wine they vary between 22.5 and 63.5 mg/L [93,94]. Wines containing more than 90 mg/L of ethyl acetate are generally considered faulty. At low concentrations, ethyl acetate enhances fruity aromas, whereas at higher levels it imparts an odor reminiscent of nail polish [57,92]. In sake fermented with wine yeasts, the ethyl acetate concentration was 5.2-fold higher for *T. delbrueckii* and 4.4-fold higher for *S. bayanus*, compared to that obtained with the conventional *S. cerevisiae* K7 strain.

In addition to ethyl acetate, wine, beer, and sake products also contain a relatively small share of other ester compounds (e.g., esters of acetic acid and higher alcohol, as well as esters of ethanol and fatty acid), which typically occur in quantities below 1 mg/L or within several mg/L. Despite their low abundance, these compounds often have extremely low sensory thresholds (in the ng/L range) and therefore have a significant impact on the overall aroma profile of wine. According to Mimura et al. [95], key contributors to the fruity aroma of sake, known as ginjo-ka, include hexanoate and octanoate esters (e.g., ethyl caproate and ethyl caprylate), along with isoamyl acetate. Among the tested yeast strains, *S. bayanus* showed the highest potential for producing this characteristic aroma. Compared to the conventional *S. cerevisiae* K7 strain, the concentrations of 3-methylbutyl acetate, 2-methylbutyl acetate, and ethyl hexanoate in sake produced with *S. bayanus* were higher by 249.5%, 590.9%, and 199.3%, respectively. No significant differences were observed in the levels of ethyl octanoate and 2-phenethyl acetate between these yeast strains.

The sensory profile imparted by *S. pastorianus* in beer is generally subtle, with esters such as ethyl acetate and isoamyl acetate serving as key contributors to its aroma. In beer, ethyl acetate typically occurs within the range of 10–60 mg/L, while isoamyl acetate is found at concentrations between 0.5 and 5 mg/L [96]. The concentrations of these compounds observed in this study for both strains of *S. pastorianus* strains during sake fermentation were within the established ranges.

According to Puligundla et al. [97], lager beers with a balanced flavor profile typically exhibit higher alcohol-to-ester ratios in the range of approximately 2.5–3:1. In the present study, this ratio was substantially higher for the brewing strains W34/70 and Diamond Lager, reaching 6.7 and 6.2, respectively. These elevated values indicate an imbalance skewed toward higher alcohol production, which is associated with a less harmonious flavor profile in lager beer. In this context, *T. delbrueckii* positively influenced the aromatic profile of sake by increasing the production of fruity esters while simultaneously lowering the concentration of higher alcohols. The resulting alcohol-to-ester ratio was 2.4, indicating a more balanced and aroma-enhancing fermentation outcome. However, this value may not be fully representative of the wine yeast strain, as the majority of esters produced were attributed to ethyl acetate, which alone may contribute to imbalances in the overall aroma profile. In contrast, this ratio exceeded 16 in the case of the sake yeast strain K7, which was characterized by very low ethyl acetate production. These findings suggest that a comprehensive sensory analysis would be essential to draw definitive conclusions regarding the impact of these metabolic differences on the final aromatic quality.

**Figure 4:**
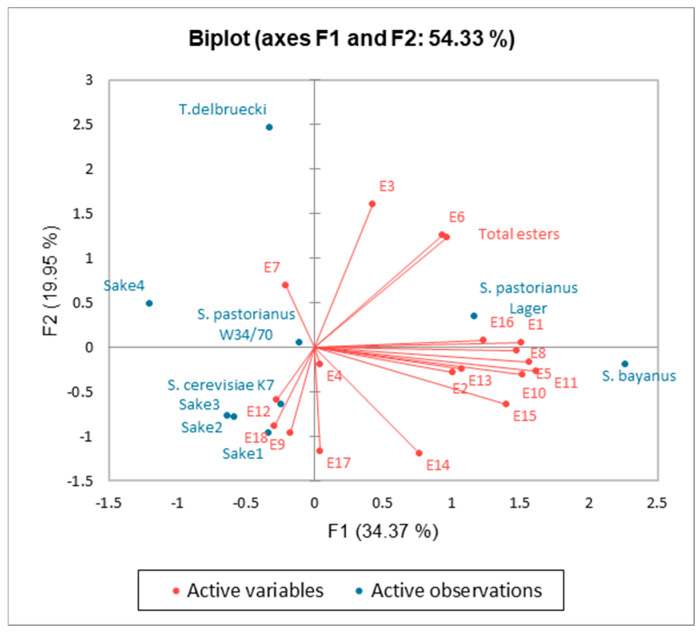
The result of PCA for the ester profile of non-conventional and commercial sake. E1: Methyl acetate, E2: n-propyl acetate, E3: Ethyl isobutyrate, E4: Butyl acetate, E5: Ethyl formate, E6: Ethyl acetate, E7: Ethyl propionate, E8: Isobutyl acetate, E9: Ethyl butyrate, E10: 3-methylbutanol acetate, E11: 2-methylbutanol acetate, E12: ethyl hexanoate, E13: Ethyl heptanoate, E14: Ethyl octanoate, E15: 2-Phenethyl acetate, E16: Ethyl nonanoate, E17: Ethyl decanoate, E18: Ethyl dodecanoate.

In the further course of the research, five commercially available sake beverages with varying alcohol contents (12.5–17.0% *v*/*v*) were subjected to gas chromatography analysis to compare the major components of the ginjo-ka aroma with those present in sake produced using non-conventional yeast strains. This aspect was further examined using principal component analysis (PCA), based on ester-type by-products of alcoholic fermentation (Figure 4). Principal components F1 and F2 accounted for 34.37% and 19.95% of the total variance, respectively. The sake samples were distributed across different quadrants of the coordinate system. Notably, three out of four commercial sake samples, along with the laboratory-scale *S. cerevisiae* K7 sake, clustered in the third quadrant, suggesting similarities in ester aroma profiles among these beverages. No distinct clustering was observed among samples produced with wine yeast and those with brewing yeast. The F1 axis showed a strong positive correlation with the total concentrations of methyl acetate, ethyl formate, isobutyl acetate, 3-methylbutanol acetate, 2-methylbutanol acetate, 2-phenethyl acetate, and ethyl nonanoate. These esters significantly influenced the aroma of sake produced with *S. bayanus*. Samples distributed along the F1 axis exhibited greater differences than those along F2, as F1 explained a larger proportion of the overall variance, indicating substantial differences in ester production between conventional sake yeast and *S. bayanus*. Factor F2 appeared to correlate with the total concentration of esters, including some of the most abundant compounds such as ethyl acetate, ethyl isobutyrate, and ethyl propionate. These compounds were associated with sake produced using *T. delbrueckii*. The PCA results revealed that wine yeasts had the greatest influence on the ester profile of the sake.

### 2.4. Color of Sake

Color is an important indicator of sake quality and product stability. High-quality sake is typically transparent, almost colorless, or exhibits only a faint straw-like tint. A shift in color toward yellow or brown in young sake may indicate oxidation, aging, or improper storage, which is regarded as a quality defect. Previous studies [98,99,100] have demonstrated that changes in color correlate with degradative processes such as photo oxidation and Maillard reactions, as well as with the presence of humic acid in brewing water. As a result of Maillard reactions, brown pigments known as melanoidins are synthetised. The extent of color change depends in part on the amino acid content. Sake such as junmai daiginjo, brewed from highly polished rice and therefore containing relatively few amino acids, shows little color change even after extended storage. In our study, potential differences in nitrogen uptake by non-conventional yeasts, along with variations in their sugar metabolism profiles, prompted a detailed investigation of color development.

The color of sake produced using non-conventional yeast strains was compared to that of conventionally fermented sake based on the total color difference value (ΔE). Parameters describing the color attributes of the samples, along with total color difference values, are presented in Table 4. Based on the results, it can be concluded that the rice wine samples were very light in color, with low saturation of yellow and green hues. No significant differences in color parameters were observed between sake produced with conventional and non-conventional yeasts. Moreover, the total color difference was small (ΔE < 1), indicating that the color differences were visually imperceptible. The use of non-conventional yeast strains in the rice wine production process did not affect the color of the final product.

### 2.5. Sensory Analysis

**Figure 5:**
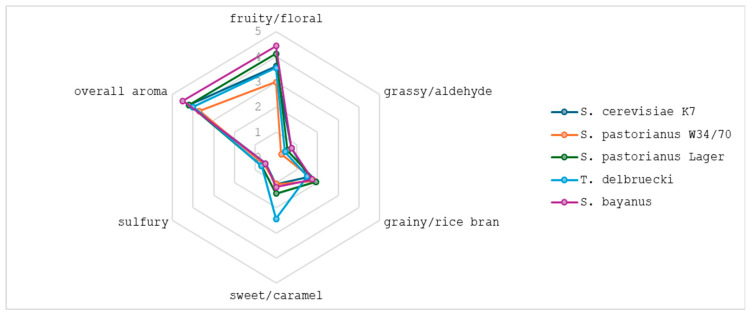
Radar plots of sensory evaluation of sake.

Non-conventional sake characteristics were explained by the sensory component profiles. Figure 5 demonstrate the mean scores of the six aroma attributes typical for sake, namely, ginjo-ka (fruity/floral), grassy/aldehyde, grainy/rice bran, sweet/caramel, sulfury, overall aroma, which were evaluated for their contribution to the aroma profile of sake. Among them, ginjo-ka was the dominating descriptor among all samples tested. Mimura et al. [95] examined the relationship between the component profile and the attributes of different sake types. Ethyl caproate was identified as the main contributor to ginjo-ka, with additional influence from hexanoate and octanoate esters. Although isoamyl acetate is commonly associated with ginjo-ka, it was not selected due to its relatively stable levels across samples, limiting its discriminative value. Our findings revealed significant variation in isoamyl acetate content across samples (Table 3). In sake fermented with *T. delbrueckii*, isoamyl acetate was the dominant ginjo-ka ester, contributing to elevated fruity/floral aroma scores. The most intense fruity and floral notes were detected in *S. bayanus*-fermented sake, surpassing conventional variants. This profile was driven by the combined presence of ethyl caproate and isoamyl acetate, whose relative proportions likely affect both aroma intensity and quality. Acetaldehyde and 3-methylbutanal, known contributors to grassy and aldehydic aromas, were most perceptible in samples fermented with *S. bayanus* and *S. cerevisiae* K7. On the other hand, *T. delbrueckii* imparted sweet and caramel-like notes to sake, likely due to its high production of 2,3-butanedione. All strains tested exhibited minimal perception of sulfur-related off-notes, typically associated with hydrogen sulfide, methyl mercaptan, and dimethyl trisulfide. Sensory evaluation confirmed that *S. bayanus* had a positive impact on wine aroma, showing the highest overall scores and the most complex flavour profile. These findings suggest that *S. bayanus* may be effectively used to enhance wine quality by improving aroma and complexity.

## 3. Materials and Methods

### 3.1. Raw Materials and Microorganisms

Yamadanishiki rice (Kobe, Japan), highly polished to 35–55%, was used in the fermentation process. Koji was prepared by inoculating the rice with *Aspergillus oryzae* spores (Koji-kin, Kensho, Nagano-ken, Japan). The yeast starters included *Saccharomyces cerevisiae* K7 (Sake #7 Yeast, White Labs, Tokyo, Japan), *Saccharomyces pastorianus* (Diamond Lager Yeast, LalBrew, Lallemand, Vienna, Austria), *S. pastorianus ssp. carlsbergensis* W34/70 (Hefebank Weihenstephan GmbH, Freising, Germany), *Torulaspora delbrueckii* (Level2 Biodiva, Lallemand, Montréal, QC, Canada), and *Saccharomyces bayanus* (Siha Aktiv Hefe 4, Lallemand, Montréal, QC, Canada).

The primary criterion for the selection of non-conventional yeasts in our study was their cryotolerance, as the moromi fermentation in sake brewing is carried out at temperatures below 15 °C. For this reason, we included bottom-fermenting brewing yeasts, which ferment efficiently at 10–15 °C, as well as sparkling wine yeasts, which are capable of conducting fermentation at 8–10 °C. In addition, *Torulaspora delbrueckii* is not specifically adapted for low-temperature fermentation, but according to the manufacturer’s specification it produces high levels of esters, which are essential contributors to the aromatic profile of sake.

Four types of commercially available sake were used as control samples: Sake 1—12.5% (*v*/*v*) (Hakutsuru, Kobe, Japan), category/style: Junmai Nigori; polishing rate: 70%. Sake 2—17.0% (*v*/*v*) (Tsuji Honten, Hiroshima, Japan), category/style: Junmai Bodaimoto Nigori; polishing rate: 65%; rice variety: Yudomachi. Sake 3—17.5% (*v*/*v*) (Takara Shuzo, Kyoto, Japan), category: Daiginjo, Muroka Genshu (Nihonshu); polishing rate: 50%; rice variety: Gohyakumangoku. Sake 4—15.0% (*v*/*v*) (Hakutsuru, Kobe, Japan), category/style: Junmai; polishing rate: 70%. For the comparative study, sake samples with varying alcohol contents were selected, ranging from light (12.5% *v*/*v*) to strong, undiluted genshu (17.5% *v*/*v*). The polishing rate ranged from 70% (yielding fuller, more rice-forward flavors) to 50% (producing a more delicate and elegant profile). The selected styles comprised: Nigori (Sake 1 and 2)—cloudy and creamy; Bodaimoto (Sake 2)—traditional and acidic; Daiginjo Muroka Genshu (Sake 3)—premium, pure, and undiluted; and Junmai (Sake 4)—classic, full-bodied, and without additives.

### 3.2. Sake Preparation

Sake was prepared following the method described by Ichikawa et al. [101], with slight modifications. Yamadanishiki rice was washed twice for 30 s using tap water at 10 °C, then drained for 1 min. The rice was subsequently soaked for 30 min to achieve a water absorption rate of 130–140%. After soaking, the rice was steamed under cover for 50 min. Following cooling, water absorption was adjusted to 130–140%, based on the assumption that the initial moisture content of the dry rice was 13.5%. The steamed rice was then inoculated with Aspergillus oryzae (Kensho preparation) at a ratio of 1 g of mold spores per 1 kg of dry rice. The mixture was incubated for 46 h at 37 °C under conditions of high humidity. Sake mash was prepared in three stages over a four-day period. A total of 55 g of rice was used in the initial stage, consisting of 11 g of koji, 44 g of steamed Yamadanishiki rice, 70 mL of water, a calculated amount of yeast inoculum (1 × 10^7^ CFU/mL), and 490 μL of 50% lactic acid. After thorough mixing, the mixture was covered with a fermentation lock and incubated at 15 °C. After 72 h, the second portion of ingredients was added to the moromi: 110 g of rice in total, comprising 22 g of koji, 88 g of steamed rice, and 140 mL of water. Incubation was continued for an additional 24 h. Subsequently, the final portion was added: 33 g of koji, 132 g of steamed rice, and 210 mL of water. Alcoholic fermentation was conducted at 10 °C. Progress of fermentation was monitored by measuring the decrease in mash mass. Upon completion, the fermented mash was centrifuged to obtain clarified sake. Our laboratory method reproduces the key stages of sake brewing (rice steaming, koji making, yeast starter, and three-step moromi fermentation). However, it is simplified in terms of scale and equipment compared with industrial production. It is important to note that reducing the scale of fermentation processes usually affects heat transfer and aeration, and also influences yeast performance and viability due to increased osmotic and hydrostatic pressure stress.

### 3.3. Basic Chemical Composition of Sake

Ethanol content and total extract were determined using the distillation method, followed by densimetric measurement with an Anton Paar DMA 5000 M densitometer (Graz, Austria). Total acidity was measured by titration. Volatile acidity was assessed following steam distillation of the sake sample and immediate titration of the distillate with standardized 0.1 M sodium hydroxide solution, using phenolphthalein as an indicator.

Amino acidity was determined by titration based on the ethanol addition method. Briefly, 10 mL of the sample were first titrated with 0.1 M sodium hydroxide to a pH of 8.2. Subsequently, 25 mL of ethanol were added to the titration mixture, and the solution was further titrated with 0.1 M sodium hydroxide until the pH reached 10.4. The volume of 0.1 M NaOH required to reach the second endpoint is defined as the amino acidity, according to the National Tax Agency of Japan [102].

### 3.4. Gas Chromatography GC/MS

The analysis of volatile b-products of alcoholic fermentation in the obtained sake was carried out using a gas chromatography apparatus (Agilent 7890 A, Santa Clara, CA, USA) with a mass spectrometer (Agilent MSD 5975C, Agilent Technologies, Santa Clara, CA, USA). The headspace autosampler operating parameters were: incubation temperature 50 °C, loop 60 °C, transfer line 70 °C, incubation time 20 min. An HP INNOWax capillary column 30 m in length, 0.25 mm film thickness, 0.4 µm i.d. was used. The oven temperature was programmed from 35 °C (7 min) to 80 °C at a rate of 5 °C/min and was then increased to 225 °C (5 min) at a rate of 10 °C/min. The flow rate of the carrier gas was 1.1 mL/min. The temperature of the injector was 250 °C. MS conditions were as follows: ion source temperature 230 °C, transfer line temperature 250 °C, and quadrupole temperature 150 °C. The ionization energy was 70 eV. Volatile compounds were quantified using calibration curves in the selected ion monitoring mode. Quantitative analysis was performed using Agilent MassHunter software (Version B.07.00/Build 7.0.457.0, Agilent Technologies, Inc. 2008, Santa Clara, CA, USA). Ethyl carbamate was extracted with dichloromethane according to the method of Mimura et al. [59]. A 5 µL aliquot of the sample was directly injected onto a TG-WaxMS column (60 m × 0.32 mm × 0.50 μm; Thermo Fisher Scientific, Waltham, MA, USA). The oven temperature was programmed from 50 °C (4 min) to 70 °C at a rate of 2 °C/min and then increased to 230 °C (7 min) at a rate of 10 °C/min. The carrier gas (helium) flow rate through the column was 1.1 mL/min. The same MS parameters as described above were used.

### 3.5. Urea and L-Arginine

The determination of ammonia, urea, and L-arginine was performed by UV spectrophotometry using the L-Arginine/Urea/Ammonia test kit (Megazyme, Bray, Ireland). The assays were performed using a Multiscan GO spectrophotometer (Thermo Fisher Scientific, Waltham, MA, USA) equipped with a microplate reader.

### 3.6. Antiradical Activity Against ABTS Radical

The radicals were generated by reaction of 7 mmol/L ABTS (2,2′-Azinobis 3-ethylbenzothiazoline-6-sulfonic acid) in water with 2.45 mmol/L potassium persulphate. Sake samples (10 µL) were mixed with 200 µL of ABTS reagent and incubated in darkness for 30 min at room temperature. Absorbance was measured at 734 nm. The assays were performed using a Multiscan GO spectrophotometer (Thermo Fisher Scientific, Waltham, MA, USA) equipped with a microplate reader. The results were presented in mmol of Trolox per 1 L of sake.

### 3.7. Color Determination Using the CIE Lab Model

The color of rice wine was determined spectrophotometrically using a Konica Minolta Chroma Meter colorimeter (Konica Minolta, Tokyo, Japan). The measurement was conducted within the CIE Lab* color space, where colors are represented as points forming a three-dimensional space defined by the L, a, and b axes. The device was calibrated using standard black and white reference tiles. Two colors were compared by calculating the total color difference, which represents the distance between two colors in the CIE Lab three-dimensional space.

### 3.8. Sensory Evaluation

Sake aroma was assessed using analytical descriptive sensory analysis. The evaluated aroma descriptors were determined according to the sake terminology system described by Utsunomiya et al. [103] with slight modifications. The panel consisted of eight trained judges with prior experience in sake evaluation. Assessors evaluated the following aroma attributes: ginjo-ka (fruity/floral), grassy/aldehyde, grainy/rice bran, sweet/caramel, sulfury, and overall aroma. A structured five-point scale was applied, ranging from weak (1) to strong (5) intensity. Samples were served in standardized sake tasting glasses at approximately 20 °C, under controlled laboratory conditions with neutral lighting and minimized external odors. Each sample was coded with a random three-digit number and presented in randomized order.

### 3.9. Statistical Analysis

Statistical analyses were carried out using XLSTAT software (version 2025.1, Addinsoft, Lumivero, France). The normality of residuals was assessed using the Shapiro–Wilk test and by visual inspection of Q–Q plots, given the known limitations of formal normality tests with small and large sample sizes. Homogeneity of variances was evaluated with Levene’s test. For all datasets analyzed, *p*-values exceeded 0.05, confirming that the assumptions for parametric analyses were satisfied. Data were then analyzed using ANOVA, followed by Tukey’s post hoc test to determine significant differences between samples. Differences were considered statistically significant at *p* < 0.05. Principal Component Analysis (PCA) was additionally performed to explore relationships between variables and samples.

## 4. Conclusions

The widespread use of K7 group strains has reduced the diversity of sake flavor profiles compared to earlier times, when breweries relied on their own distinctive house yeast strains. Although K7 group strains possess excellent characteristics for sake production, increased use of other yeast strains with distinctive sensory profiles may diversify the organoleptic characteristics of sake and expand the range of sake categories. This study demonstrated that non-conventional strains can be used in sake brewing to produce unique products. Sake produced using commercial brewing and wine yeast strains differed in fermentation kinetics, chemical composition, and sensory properties. Yeast and fermentation conditions are considered the primary factors influencing wine flavour.

Our findings suggest that the wine yeast *S. bayanus* can be considered valuable in sake production technology. This is due to its high production of volatile compounds responsible for the Ginjo-ka aroma qualities of the final product, as well as its ability to support a proper alcoholic fermentation process. The results obtained in this study may represent a valuable contribution to the development of innovative sake production technologies aimed at achieving high sensory quality.

The non-conventional sake obtained in this study may be classified as low-alcohol, as its ABV level is below 13%, whereas traditional sake typically contains more than 15% alcohol. Low-alcohol beverages represent a rapidly growing sector, driven largely by the shift toward healthier lifestyles. According to the International Wine and Spirits Record (IWSR), across 10 key markets (Australia, Brazil, Canada, France, Germany, Japan, Spain, South Africa, the UK, and the US), the combined no/low-alcohol market is expected to expand by +4% volume CAGR through 2028 [104]. The low- and non-alcoholic beer sector has long driven the trend toward LNA beverages, and its success later inspired the introduction of low- and non-alcoholic wine and spirits. Entering the LNA market is therefore an important strategy for producers seeking to diversify their product portfolios and sustain competitiveness [105]. In this context, the prospects for low-alcohol sake production also appear promising. With overseas markets expanding, sake is undergoing a process of redefinition evolving into a lighter, more inclusive, and experimental beverage that resonates with contemporary tastes and values.

Our future research on reduced-alcohol sake will focus particularly on its aging potential. Most low-alcohol styles are designed for early, fresh consumption rather than for long-term maturation. A lower ethanol concentration generally results in reduced shelf stability and a greater susceptibility to undesirable changes in the bottle, such as microbial spoilage or the development of off-flavors. In this context, future innovation should not aim to replicate the long-aging tradition of full-strength sake, but rather to define new strategies that achieve complexity, stability, and consumer appeal within the unique constraints of the low-alcohol category.

## Figures and Tables

**Table 1:** Basic chemical composition and antiradical activity of sake.

	*S. cerevisiae K7*	*S. pastorianus* W34/70	*S. pastorianus* Lager	*T. delbruecki* *i*	*S. bayanus*
Alcohol [% (*v*/*v*)]	11.6 ± 0.3 b	11.8 ± 0.0 b	10.7 ± 0.1 c	11.1 ± 0.1 bc	12.3 ± 0.4 a
Total extract [g/L]	22.14 ± 0.00 a	21.13 ± 0.10 b	20.12 ± 0.00 c	23.23 ± 0.42 d	18.09 ± 0.00 e
Total acidity [g/L]	1.87 ± 0.05 bc	1.83 ± 0.09 c	1.94 ± 0.12 b	2.42 ± 0.07 a	1.95 ± 0.04 b
Volatile acidity [g/L]	0.39 ± 0.04 a	0.33 ± 0.03 a	0.36 ± 0.00 a	0.54 ± 0.04 b	0.50 ± 0.00 b
Amino acidity [mL]	2.55 ± 0.07 a	2.45 ± 0.07 ab	2.05 ± 0.06 c	2.20 ± 0.00 bc	2.05 ± 0.07 c
Free SO_2_ [mg/L]	2.74 ± 0.09 ab	2.83 ± 0.24 a	2.34 ± 0.21 b	2.33 ± 0.25 b	2.30 ± 0.15 b
Total SO_2_ [mg/L]	13.49 ± 0.55 b	13.73 ± 0.41 ab	13.67 ± 0.55 ab	13.94 ± 0.38 ab	14.07 ± 0.70 a
Antiradical activity [mM/L]	0.32 ± 0.04 a	0.33 ± 0.01 a	0.25 ± 0.01 a	0.23 ± 0.01 a	0.29 ± 0.02 a

Results are expressed as mean ± standard deviation (n = 3). ANOVA assumptions were verified (Shapiro–Wilk test for normality, *p* > 0.05; Levene’s test for homogeneity of variances, *p* > 0.05). Different letters within the same row indicate statistically significant differences between sake variants (ANOVA, *p* < 0.05).

**Table 2:** Concentration of ethyl carbamate and its precursors in sake.

	*S. cerevisiae K7*	*S. pastorianus* W34/70	*S. pastorianus* Lager	*T. delbrueckii*	*S. bayanus*
Arginine [mg/L]	nd	nd	nd	nd	nd
Urea [mg/L]	6.62 ± 0.28 a	4.23 ± 0.20 b	5.80 ± 0.27 ab	6.25 ± 0.32 a	4.42 ± 0.17 b
Ethyl carbamate LOD 4.89 µg/L LOQ 14.82 µg/L	nd	nd	nd	nd	nd

nd—not detected. Results are expressed as mean ± standard deviation (n = 3). ANOVA assumptions were verified (Shapiro–Wilk test for normality, *p* > 0.05; Levene’s test for homogeneity of variances, *p* > 0.05). Different letters within the same row indicate statistically significant differences between sake variants (ANOVA, *p* < 0.05).

**Table 3:** Qualitative and quantitative composition of volatile compounds in sake (mg/L).

Compound	*S. cerevisiae* K7		*S. pastorianus* W34/70		*S. pastorianus* Lager		*T. delbrueckii*		*S. bayanus*	
	Mean	±SD	Mean	±SD	Mean	±SD	Mean	±SD	Mean	±SD
Acetaldehyde	51.125 ab	3.935	33.149 c	0.577	39.680 bc	2.478	25.122 c	6.382	61.992 a	0.914
Methyl acetate	0.009 c	0.001	0.033 b	0.005	0.072 a	0.004	0.011	0.002	0.058 ac	0.006
1-Propanol	46.187 bc	2.448	35.613 dc	1.600	51.006 b	4.614	27.837 d	3.542	68.962 a	1.215
n-Propyl acetate	0.013 b	0.000	0.015 b	0.001	0.035 b	0.007	0.023 b	0.001	0.092 ac	0.009
Ethyl isobutyrate	0.003 d	0.000	0.008 cd	0.000	0.017 b	0.002	0.032 a	0.001	0.009	0.000
Butyl acetate	0.000 c	0.000	0.002 a	0.000	0.001 b	0.000	0.000 c	0.000	0.000 c	0.000
Ethyl formate	0.149 b	0.017	0.159 b	0.014	0.139 b	0.032	0.032 c	0.014	0.523 a	0.009
Isobutyraldehyde	0.182 bc	0.009	0.133 cd	0.003	0.200 b	0.019	0.106 d	0.011	0.270 a	0.004
2,3-Butanedione	0.011 a	0.002	0.008 a	0.001	0.013 a	0.002	0.074 a	0.040	0.011 a	0.001
Ethyl acetate	17.372 d	1.231	36.980 c	1.072	57.139 b	6.219	90.869 a	3.653	76.712 a	3.916
Isobutyl alcohol	82.345 ab	4.253	55.023 b	2.724	97.395 a	9.859	57.951 b	7.633	93.051 a	3.181
3-Methylbutanal	0.041 a	0.002	0.026 b	0.001	0.041 a	0.004	0.017 b	0.003	0.047 a	0.002
2-Methylbutanal	0.006 b	0.000	0.004 c	0.000	0.008 a	0.000	0.002 d	0.000	0.009 a	0.000
Ethyl propionate	0.021 d	0.000	0.159 b	0.015	0.178 b	0.010	0.223 a	0.004	0.099 c	0.001
Acetal	1.022 b	0.083	0.604 c	0.027	0.613 c	0.041	0.417 c	0.109	1.450 a	0.009
3-Methylbutanol	118.207 b	1.860	122.088 b	2.743	150.481 a	3.266	95.574 c	4.434	113.396 b	3.027
2-Methylbutanol	30.401 bc	0.311	28.138 c	0.804	50.189 a	3.191	23.578 c	2.384	39.228 b	0.657
Isobutyl acetate	0.032 b	0.000	0.026 b	0.001	0.064 b	0.008	0.048 b	0.015	0.187 a	0.033
Ethyl butyrate	0.085 b	0.005	0.074 b	0.001	0.107 b	0.012	0.077 b	0.005	0.195 a	0.021
3-Methylbutanol acetate	0.441 c	0.019	0.674 bc	0.008	1.340 a	0.117	0.165 c	0.057	1.100 ab	0.185
2-Methylbutanol acetate	0.023 b	0.001	0.027 b	0.001	0.075 b	0.007	0.022 b	0.009	0.137 a	0.023
Ethyl hexanoate	0.066 b	0.001	0.058 b	0.004	0.085 b	0.012	0.011 c	0.002	0.131 a	0.011
2-Phenylethanol	20.488 a	5.105	18.562 a	2.282	23.698 a	2.287	18.358 a	0.535	27.261 a	5.639
Ethyl octanoate	0.070 ab	0.002	0.055 b	0.010	0.070 ab	0.004	0.003 c	0.001	0.098 a	0.011
2-Phenethyl acetate	0.098 ab	0.001	0.056 b	0.002	0.100 ab	0.015	0.051 b	0.004	0.120 a	0.016
Ethyl decanoate	0.004 a	0.000	0.006 a	0.001	0.005 a	0.001	0.000 a	0.000	0.005 a	0.000

Results are expressed as mean ± standard deviation (n = 3). ANOVA assumptions were verified (Shapiro–Wilk test for normality, *p* > 0.05; Levene’s test for homogeneity of variances, *p* > 0.05). Different letters within the same row indicate statistically significant differences between sake variants (ANOVA, *p* < 0.05).

**Table 4:** Parameters of sake color.

	L	a*	b*	ΔE
*S. cerevisiae K7*	96.26 ± 0.12 a	−0.44 ± 0.04 ab	2.70 ± 0.06 a	-
*S. pastorianus* W34/70	96.22 ± 0.12 a	−0.44 ± 0.04 ab	2.61 ± 0.12 a	0.15 ± 0.04
*S. pastorianus* Diamond Lager	96.24 ± 0.04 a	−0.36 ± 0.02 a	2.63 ± 0.09 a	0.12 ± 0.06
*T. delbruecki* *i*	96.12 ± 0.20 a	−0.36 ± 0.01 a	2.66 ± 0.15 a	0.23 ± 0.09
*S. bayanus*	96.27 ± 0.13 a	−0.48 ± 0.02 b	2.79 ± 0.04 a	0.14 ± 0.01

Results are expressed as mean ± standard deviation (n = 3). ANOVA assumptions were verified (Shapiro–Wilk test for normality, *p* > 0.05; Levene’s test for homogeneity of variances, *p* > 0.05). Different letters within the same column indicate statistically significant differences between sake variants (ANOVA, *p* < 0.05).

## Data Availability

Additional raw data can be obtained through contact with the corresponding author.

## References

[1] Yoshizawa K. (1999). Sake: Production and flavour. Food Rev. Int..

[2] Fleet G.H. (2003). Yeast interactions and wine flavour. Int. J. Food Microbiol..

[3] Takemitsu H., Amako M., Sako Y., Shibakusa K., Kita K., Kitamura S., Inui H. (2016). Analysis of Volatile Odor Components of Superheated Steam-Cooked Rice with a Less Stale Flavor. Food Sci. Technol. Res..

[4] Yoshizaki Y., Furusawa H., Okutsu K., Hashizume K., Tanaka T., Hoshino T., Iwashita K. (2010). Analysis of volatile compounds in shochu koji, sake koji, and steamed rice by GC–MS. J. Inst. Brew..

[5] Verma D.K., Srivastav P.P., Verma D.K., Srivastav P.P. (2018). Introduction to Rice Aroma, Flavor, and Fragrance. Science and Technology of Aroma, Flavor, and Fragrance in Rice.

[6] Zhang K., Wu W., Yan Q. (2020). Research advances on sake rice, koji, and sake yeast: A review. Food Sci. Nutr..

[7] Katou T., Namise M., Kitagaki H., Akao T., Shimoi H. (2009). QTL mapping of sake brewing characteristics of yeast. J. Biosci. Bioeng..

[8] Kitagaki H., Kitamoto K. (2013). Breeding research on sake yeasts in Japan: History, recent technological advances, and future perspectives. Annu. Rev. Food Sci. Technol..

[9] Takao Y., Takahashi T., Yamada T., Goshima T., Isogai A., Sueno K., Fujii T., Akao T. (2018). Characteristic features of the unique house sake yeast strain *Saccharomyces cerevisiae* Km67 used for industrial sake brewing. J. Biosci. Bioeng..

[10] Yamasaki R., Goshima T., Oba K., Kanai M., Ohdoi R., Hirata D., Akao T. (2020). Development of sake yeast haploid set with diverse brewing properties using sake yeast strain Hiroshima no. 6 exhibiting sexual reproduction. J. Biosci. Bioeng..

[11] Suto M., Kawashima H. (2022). Discrimination for sake brewing methods by compound-specific isotope analysis and formation mechanism of organic acids in sake. Food Chem..

[12] Baba S., Hamasaki T., Sawada K., Orita R., Nagano Y., Kimura K., Goto M., Kobayashi G. (2021). Breeding sake yeast and identification of mutation patterns by synchrotron light irradiation. J. Biosci. Bioeng..

[13] Takahashi T., Ohara Y., Sawatari M., Sueno K. (2017). Isolation and characterization of sake yeast mutants with enhanced isoamyl acetate productivity. J. Biosci. Bioeng..

[14] Takahashi T., Ohara Y., Sueno K. (2017). Breeding of a sake yeast mutant with enhanced ethyl caproate productivity in sake brewing using rice milled at a high polishing ratio. J. Biosci. Bioeng..

[15] Ikeda Y., Isogai A., Moriyoshi Y., Kanda R., Iwashita K., Fujii T. (2018). Construction of sake yeast with low production of dimethyl trisulfide precursor by a self-cloning method. J. Biosci. Bioeng..

[16] Fukuda K., Watanabe M., Asano K., Ouchi K., Takasawa S. (1991). Isolation and genetic study of p-fluoro-DL-phenylalanine-resistant mutants overproducing β-phenethyl-alcohol in *Saccharomyces cerevisiae*. Curr. Genet..

[17] Fukuda K., Watanabe M., Asano K., Ueda H., Ohta S. (1990). Breeding of brewing yeast producing a large amount of β-phenylethyl alcohol and β-phenylethyl acetate. Agric. Biol. Chem..

[18] Ouchi K., Akiyama H. (1971). Non-foaming mutants of sake yeasts selection by cell agglutination method and by froth flotation method. Agric. Biol. Chem..

[19] Shimoi H., Sakamoto K., Okuda M., Atthi R., Iwashita K., Ito K. (2002). The *AWA1* gene is required for the foam-forming phenotype and cell surface hydrophobicity of sake yeast. Appl. Environ. Microbiol..

[20] Kuribayashi T., Sato K., Joh T., Kaneoke M. (2017). Breeding of a non-urea-producing sake yeast carrying a *FAS2* mutation. Mycoscience.

[21] Hara M., Sasaki M., Obata T., Nojiro K. (1976). Isolation of an ethanol-resistant strain from sake yeast Kyokai no. 7. J. Brew. Soc. Jpn..

[22] Aikawa M., Suizu T., Ichikawa E., Kawato A., Abe Y., Imayasu S. (1992). Breeding of higher malic acid–productive mutants from *Saccharomyces cerevisiae* Kyokai no. 7 (K-7). Hakkokogaku Kaishi.

[23] Asano T., Kurose N., Tarumi S. (2001). Isolation of high-malate-producing sake yeasts from low-maltose-assimilating mutants. J. Biosci. Bioeng..

[24] Kitagaki H., Kato T., Isogai A., Mikami S., Shimoi H. (2008). Inhibition of mitochondrial fragmentation during sake brewing causes high malate production in sake yeast. J. Biosci. Bioeng..

[25] Koseki T., Kudo S., Matsuda Y., Ishigaki H., Ajiki Y., Muraoka Y., Wada Y. (2004). Tyrosol High-Productivity Yeast Mutant and Method for Producing Fermented Alcoholic Beverage Using the Mutant. Jap. Open Pat. Gaz..

[26] Soejima H., Tsuge K., Yoshimura T., Koganemaru K., Kitagaki H. (2012). Breeding of a high tyrosol-producing sake yeast by isolation of an ethanol-resistant mutant from *trp3* mutant. J. Inst. Brew..

[27] Yamada T., Furukawa K., Hara S., Mizoguchi H. (2005). Isolation of copper-tolerant mutants of sake yeast with defective peptide uptake. J. Biosci. Bioeng..

[28] Akao T., Yashiro I., Hosoyama A., Kitagaki H., Horikawa H., Watanabe D., Akada R., Ando Y., Harashima S., Inoue T. (2011). Whole-genome sequencing of sake yeast *Saccharomyces cerevisiae* Kyokai no. 7. DNA Res..

[29] Kanai M., Shibata T., Zhou Y., Hayashi R., Fukuba I., Kochi T., Teramoto S., Shimoi H., Takahashi H., Akao T. (2025). Efficient gene identification via quantitative trait loci analysis by crossbreeding of sake and laboratory yeast. Appl. Microbiol. Biotechnol..

[30] Chadani T., Ohnuki S., Isogai A., Goshima T., Kashima M., Ghanegolmohammadi F., Nishi T., Hirata D., Watanabe D., Kitamoto K. (2021). Genome editing to generate sake yeast strains with eight mutations that confer excellent brewing characteristics. Cells.

[31] Hirosawa I., Aritomi K., Hoshida H., Kashiwagi S., Nishizawa Y., Akada R. (2004). Construction of a self-cloning sake yeast that overexpresses alcohol acetyltransferase gene by a two-step gene replacement protocol. Appl. Microbiol. Biotechnol..

[32] Nakagawa T., Yoshimura A., Sawai Y., Hisamatsu K., Akao T., Masaki K. (2024). Japanese sake making using wild yeasts isolated from natural environments. Biosci. Biotechnol. Biochem..

[33] Varela C. (2016). The impact of non-*Saccharomyces* yeasts in the production of alcoholic beverages. Appl. Microbiol. Biotechnol..

[34] Benito S., Ruiz J., Belda I., Kiene F., Beisert B., Navascués E., Marquina D., Calderón F., Santos A., Rauhut D., Sibirny A. (2019). Application of non-*Saccharomyces* yeasts in wine production. Non-conventional Yeasts: From Basic Research to Application.

[35] Sannino C., Mezzasoma A., Buzzini P., Turchetti B., Sibirny A. (2019). Non-conventional yeasts for producing alternative beers. Non-conventional Yeasts: From Basic Research to Application.

[36] Dashko S., Zhou N., Compagno C., Piškur J. (2014). Why, When, and How Did Yeast Evolve Alcoholic Fermentation?. Appl. Environ. Microbiol..

[37] Walker G.M. (2016). Yeast Physiology and Biotechnology. Beverages.

[38] Roca-Mesa H., Sendra S., Mas A., Portillo M.C. (2020). Nitrogen Preferences during Alcoholic Fermentation of Non-Saccharomyces Wine Yeasts. Microorganisms.

[39] Crépin L., Nidelet T., Sanchez I., Dequin S., Camarasa C. (2012). Sequential Use of Nitrogen Compounds by Saccharomyces cerevisiae during Wine Fermentation: A Model Based on Kinetic and Regulation Characteristics of Nitrogen Permeases. Appl. Environ. Microbiol..

[40] Sahana T.G., Ganesan K., Venugopal V., Rajendran R., Xu J., Balasubramanian G. (2024). Mechanisms of Ethanol Tolerance in Yeast: A Review. Microb. Cell Fact..

[41] Riles L., Fay J.C. (2018). Genetic Basis of Variation in Saccharomyces cerevisiae Sensitivity to Ethanol Stress. G3 Genes|Genomes|Genetics.

[42] Bideaux C., Alfenore S., Cameleyre X., Molina-Jouve C., Guillouet S.E. (2006). Short-Term Dynamics of Glycerol Production in Saccharomyces cerevisiae. FEMS Yeast Res..

[43] Navarrete C., Treu L., Campanaro S., da Silva L.V., Quirós M., Lopandić K., Ciani M., Pretorius I.S., Dequin S., Morales P. (2014). Metabolic Engineering of Glycerol Production in Saccharomyces cerevisiae for Industrial Applications. AMB Express.

[44] de Smidt O., du Preez J.C., Albertyn J. (2008). The Alcohol Dehydrogenases of Saccharomyces cerevisiae: A Comprehensive Review. FEMS Yeast Res..

[45] Nakatani M., Ohtani R., Umezawa K., Uchise T., Matsuo Y., Fukuta Y., Obata E., Katabuchi A., Kizaki K., Kitazume H. (2024). Characterization and application of *Lachancea thermotolerans* isolates for sake brewing. J. Biosci. Bioeng..

[46] Yamaguchi S. (1991). Basic properties of umami and effects on humans. Physiol. Behav..

[47] Schmidt C.V., Olsen K., Mouritsen O.G. (2021). Umami potential of fermented beverages: Sake, wine, champagne, and beer. Food Chem..

[48] Okada K., Gogami Y., Oikawa T. (2013). Principal component analysis of the relationship between the D-amino acid concentrations and the taste of the sake. Amino Acids.

[49] Iwano K., Takahashi K., Ito T., Nakazawa N. (2004). Search for amino acids affecting the taste of Japanese sake. J. Brew. Soc. Jpn..

[50] El Hosry L., Elias V., Chamoun V., Halawi M., Cayot P., Nehme A., Bou-Maroun E. (2025). Maillard reaction: Mechanism, influencing parameters, advantages, disadvantages, and food industrial applications—A review. Foods.

[51] Boerzhijin S., Isogai A., Mukai N. (2023). Impact of storage conditions on the volatile aroma compounds of aged sake. J. Food Compos. Anal..

[52] Isogai A., Utsunomiya H., Kanda R., Iwata H. (2005). Changes in the aroma compounds of sake during aging. J. Agric. Food Chem..

[53] Swiegers J., Pretorius I. (2007). Modulation of volatile sulfur compounds by wine yeast. Appl. Microbiol. Biotechnol..

[54] Mendoza-Cózatl D., Loza-Tavera H., Hernández-Navarro A., Moreno-Sánchez R. (2005). Sulfur assimilation and glutathione metabolism under cadmium stress in yeast, protists and plants. FEMS Microbiol. Rev..

[55] Belda I., Ruiz J., Esteban-Fernández A., Navascués E., Marquina D., Santos A., Moreno-Arribas M.V. (2017). Microbial contribution to wine aroma and its intended use for wine quality improvement. Molecules.

[56] Ogata K. (2013). Hydrogen sulphide production by bottom-fermenting yeast is related to nitrogen starvation signalling. J. Inst. Brew..

[57] Zhang H., Cui Y. (2023). Study of sulphides production affecting the fermentation process of lager beer. Int. J. Biol. Life Sci..

[58] Andorrà I., Martín L., Nart E., Puxeu M., Hidalgo C., Ferrer-Gallego R. (2018). Effect of grape juice composition and nutrient supplementation on the production of sulfur dioxide and carboxylic compounds by Saccharomyces cerevisiae. Aust. J. Grape Wine Res..

[59] Wells A., Osborne J.P. (2011). Production of SO_2_ binding compounds and SO_2_ by Saccharomyces during alcoholic fermentation and the impact on malolactic fermentation. S. Afr. J. Enol. Vitic..

[60] (2021). Regulation (EU) No 2021/2117 of the European Parliament and of the Council of 2 December 2021 amending Regulations (EU) No 1308/2013, (EU) No 1151/2012, (EU) No 251/2014 and (EU) No 228/2013. Off. J. Eur. Union.

[61] (2013). Regulation (EU) No 1308/2013 of the European Parliament and of the Council of 17 December 2013 establishing a common organisation of the markets in agricultural products. Off. J. Eur. Union.

[62] Abe Y., Saito T., Okamoto T., Sasaki T., Hoshi Y., Sugie M., Hagiwara S., Shiga T. (2012). Investigation of changes in antioxidant activity during sake brewing by the ORAC method. J. Brew. Soc. Jpn..

[63] Kitagaki H., Tsugawa M. (1999). 1,1-Diphenyl-2-picrylhydrazyl radical (DPPH) scavenging ability of sake during storage. J. Biosci. Bioeng..

[64] Tsuji A., Koyanagi T. (2025). Significant contribution of amino acids to antioxidant capacity of Japanese rice wine (sake). J. Sci. Food Agric..

[65] IARC Working Group on the Evaluation of Carcinogenic Risks to Humans (2010). Alcohol Consumption and Ethyl Carbamate. IARC Monographs on the Evaluation of Carcinogenic Risks to Humans.

[66] Pflaum T., Hausler T., Baumung C., Ackermann S., Kuballa T., Rehm J., Lachenmeier D.W. (2016). Carcinogenic compounds in alcoholic beverages: An update. Arch. Toxicol..

[67] Ough C.S., Crowell E.A., Gutlove B.R. (1988). Carbamyl compound reactions with ethanol. Am. J. Enol. Vitic..

[68] Abt E., Incorvati V., Posnick R.L., Redan B.W. (2021). Occurrence of ethyl carbamate in foods and beverages: Review of the formation mechanisms, advances in analytical methods, and mitigation strategies. J. Food Prot..

[69] Kim Y.G., Lyu J., Kim M.K., Lee K.-G. (2015). Effect of citrulline, urea, ethanol, and urease on the formation of ethyl carbamate in soybean paste model system. Food Chem..

[70] Wang P., Sun J., Li X., Wu D., Li T., Lu J., Chen J., Xie G. (2014). Contribution of citrulline to the formation of ethyl carbamate during Chinese rice wine production. Food Addit. Contam. A.

[71] Stewart G.G. (2016). *Saccharomyces* species in the production of beer. Beverages.

[72] Fu M.-L., Liu J., Chen Q.-H., Liu X.-J., He G.-Q., Chen J.-C. (2010). Determination of Ethyl Carbamate in Chinese Yellow Rice Wine Using High-Performance Liquid Chromatography with Fluorescence Detection. Int. J. Food Sci. Technol..

[73] Wu Q., Cui K., Lin J., Zhu Y., Xu Y. (2017). Urea production by yeasts other than *Saccharomyces* in food fermentation. FEMS Yeast Res..

[74] Benito Á., Jeffares D., Palomero F., Calderón F., Bai F.-Y., Bähler J., Benito S. (2016). Selected *Schizosaccharomyces pombe* strains have characteristics that are beneficial for winemaking. PLoS ONE.

[75] Larcher R., Moser S., Menolli A.U., Tonidandel L., Nicolini G. (2013). Ethyl carbamate formation in sub-optimal wine storage conditions and influence of the yeast starter. J. Int. Sci. Vigne Vin.

[76] Fang F., Qiu Y., Du G., Chen J. (2018). Evaluation of ethyl carbamate formation in Luzhou-flavor spirit during distillation and storage processes. Food Biosci..

[77] Leça J.M., Pereira V., Miranda A., Vilchez J.L., Marques J.C. (2021). New insights into ethyl carbamate occurrence in fortified wines. LWT-Food Sci. Technol..

[78] Liu X., Qian M., Dong H., Bai W., Zhao W., Li X., Liu G. (2020). Effect of ageing process on carcinogen ethyl carbamate (EC), its main precursors and aroma compound variation in Hakka Huangjiu produced in southern China. Int. J. Food Sci. Technol..

[79] Ministry of Health, Labour and Welfare (Japan) (2020). Food Sanitation Act.

[80] Ministry of Agriculture, Forestry and Fisheries (Japan) (2022). Japanese Agricultural Standards (JAS).

[81] National Tax Agency (Japan) (1989). Standards for Sake Production and Labeling (Notification No. 8 of 1989).

[82] Health Canada (2020). Health Canada’s Maximum Levels for Chemical Contaminants in Foods.

[83] Heller L. Canada Approves GM Yeast That Combats Cancer Compound. https://www.beveragedaily.com/Article/2006/10/06/Canada-approves-GM-yeast-that-combats-cancer-compound/.

[84] Coulon J., Husnik J.I., Inglis D.L., van der Merwe G.K., Lonvaud A., Erasmus D.J., van Vuuren H.J.J. (2006). Metabolic engineering of *Saccharomyces cerevisiae* to minimize the production of ethyl carbamate in wine. Am. J. Enol. Vitic..

[85] OIV (2022). International Code of Oenological Practices.

[86] Huang D., Zhong Y., Liu Y.L., Song Y.Y., Zhao X.X., Qin Y. (2023). Reducing higher alcohols by integrating indigenous *Saccharomyces cerevisiae*, nitrogen compensation, and chaptalization methods during fermentation of kiwifruit wine. LWT-Food Sci. Technol..

[87] Fikselová K., Mátéová K., Špánik I. (2017). Effect of indigenous *S. cerevisiae* strains on higher alcohols, volatile acids and esters in wine. Czech J. Food Sci..

[88] Belda I., Navascués E., Marquina D., Santos A., Calderón F., Benito S. (2015). Dynamic analysis of physiological properties of *Torulaspora delbrueckii* in wine fermentations and its incidence on wine quality. Appl. Microbiol. Biotechnol..

[89] Azzolini M., Tosi E., Lorenzini M., Finato F., Zapparoli G. (2015). Contribution to the aroma of white wines by controlled *Torulaspora delbrueckii* cultures in association with *Saccharomyces cerevisiae*. World J. Microbiol. Biotechnol..

[90] Chen L., Ren X., Wang Y., Hao D., Liang Y., Qin Y. (2025). Transcriptomic identification of core regulatory genes for higher alcohol production in *Saccharomyces cerevisiae* at different sugar concentrations in wine fermentation. Foods.

[91] Yang H., Wang X., Liu P., Niu H., Wang L., Wang Z. (2023). Research progress of wine aroma components: A critical review. Food Chem..

[92] Lambrechts M.G., Pretorius I.S. (2000). Yeast and its importance to wine aroma—A review. S. Afr. J. Enol. Vitic..

[93] Swiegers J.H., Pretorius I.S. (2005). Yeast modulation of wine flavor. Adv. Appl. Microbiol..

[94] Saerens S.M.G., Delvaux F.R., Verstrepen K.J., Thevelein J.M. (2010). Production and biological function of volatile esters in *Saccharomyces cerevisiae*. Microb. Biotechnol..

[95] Mimura N., Isogai A., Iwashita K., Bamba T., Fukusaki E. (2014). Gas chromatography/mass spectrometry based component profiling and quality prediction for Japanese sake. J. Biosci. Bioeng..

[96] Lin C., de la Cerda García-Caro R., Zhang P., Carlin S., Gottlieb A., Petersen M., Vrhovsek U., Bond U. (2021). Packing a punch: Understanding how flavours are produced in lager fermentations. FEMS Yeast Res..

[97] Puligundla P., Smogrovicova D., Mok C., Obulam V.S.R. (2020). Recent developments in high gravity beer-brewing. Innov. Food Sci. Emerg. Technol..

[98] Satō S., Nakamura K., Tadenuma M., Yoda M., Koseki T., Hamachi M. (1971). Studies on Changes in Sake Caused by Light and Storage (Part XI). Formation of Kynurenic Acid by Yeast. J. Soc. Brew. Jpn..

[99] Nishidono Y., Misaka S., Maejima Y., Shimomura K., Tanaka K. (2023). Comparative Analysis of Functional Components in Sakekasu (Sake Lees). Funct. Foods Health Dis..

[100] Nose A., Hamasaki T., Hojo M. (2015). Effect of Humic Acid in Water on the Coloring of Japanese Sakes. J. Brew. Soc. Jpn..

[101] Ichikawa E., Hirata S., Hata Y., Yazawa H., Tamura H., Kaneoke M., Iwashita K., Hirata D. (2019). Analysis of metabolites in Japanese alcoholic beverage sake made from the rice Koshitanrei. J. Biosci. Bioeng..

[102] Kotani A., Watanabe J., Machida K., Yamamoto K., Hakamata H. (2022). Determination of amino acidity in Japanese sake based on the voltammetric measurement of surplus acid by quinone reduction. Electrochemistry.

[103] Utsunomiya H., Isogai A., Iwata H., Nakano S. (2006). Flavor terminology and reference standards for sensory analysis of sake. Rep. Res. Inst. Brew..

[104] https://www.theiwsr.com/insight/growth-of-4bn-expected-from-no-alcohol-category-by-2028/.

[105] Agra CEAS Consulting S.A., Areté s.r.l., Directorate-General for Agriculture and Rural Development (European Commission) (2023). Study on low/no alcohol beverages—Final report, Publications Office of the European Union. https://data.europa.eu/doi/10.2762/315469.

